# Stone-induced urethral fistula treatment with microfragmented adipose tissue containing mesenchymal stem cells: a case report from veterinary medicine with potential application in humans

**DOI:** 10.3325/cmj.2024.65.288

**Published:** 2024-06

**Authors:** Tugomir Karadjole, Ivan Butković, Ana Dimova, Vilim Molnar, Juraj Šavorić, Goran Bačić, Dragan Primorac

**Affiliations:** 1 Clinic for Reproduction and Obstetrics, Faculty of Veterinary Medicine University of Zagreb, Zagreb, Croatia; 2 St. Catherine Specialty Hospital, Zagreb, Croatia; 3 School of Medicine, Josip Juraj Strossmayer University of Osijek, Osijek, Croatia; 4 Medical School, University of Rijeka, Rijeka, Croatia; 5 Medical School, University of Split, Split, Croatia; 6 Faculty of Dental Medicine and Health, Josip Juraj Strossmayer University of Osijek, Osijek, Croatia; 7 Eberly College of Science, The Pennsylvania State University, State College, PA, USA; 8 University of New Haven, Henry C. Lee College of Criminal Justice and Forensic Sciences, West Haven, CT, USA; 9 REGIOMED Kliniken, Coburg, Germany; Karadjole et al: Stone-induced urethral fistula treatment with microfragmented adipose tissue containing MSCs; Correspondence to: Dragan Primorac St. Catherine Specialty Hospital Branimirova 71E 10000 Zagreb, Croatia dragan.primorac@svkatarina.hr

## Abstract

We report on a case of a two-year-old male dog, breed chow-chow, who suffered from urethral fistula as a result of ureterolithiasis. The urethral defect was identified intraoperatively with methylene blue. An autologous regenerative approach was combined with surgical closure of the defect, due to the well-known healing issues of the urethral wall in such conditions. A part of abdominal fat tissue was dissected to produce microfragmented adipose tissue containing mesenchymal stem cells, which was combined with platelet-rich plasma. The final product was applied in the area around the urethral defect closure. One month after the procedure, healing was confirmed with positive-contrast cystography. This therapeutic approach yielded success, and the follow-up period of one year was uneventful. The observed positive outcome of this approach in the canine model may be considered as a starting point for investigating the translational potential of the treatment in human medicine.

Urethral obstruction in canines is a complex and potentially life-threatening condition that requires timely and effective intervention. It is caused by bladder stones, urethral stones, malignancies, or prostate diseases, and can lead to significant discomfort and systemic complications. Symptoms associated with urethral obstruction, including discomfort during urination, frequent urination, blood in the urine, vomiting, lethargy, and loss of appetite, highlight the severity and immediate impact of this condition on canine health (1). Calculi or uroliths are assembled crystalloids combined with an organic matrix (2). The precise location of obstructions and any associated damage to the urinary system are identified by diagnostic imaging, which is crucial for planning the surgical or medical management (3). After the failure of catheterization, the surgical approach is indicated (4). The challenges of treating such conditions are compounded by the high risk of postoperative complications, such as strictures and fistulas, which necessitate innovative approaches to enhance healing and reduce recurrence (5). The incorporation of regenerative medicine techniques in treating such conditions promises to minimize complications and optimize clinical outcomes by harnessing the body's own healing mechanisms. We present a case of a canine patient with a stone-induced urethral fistula that was treated with microfragmented adipose tissue (MFAT) (6) containing mesenchymal stem cells (MSCs) and platelet-rich plasma (PRP) in addition to surgical repair. The observed positive outcome of this approach in the canine model may be considered as a starting point for investigating the translational potential of the treatment in human medicine.

## Case report

**Figure 1:**
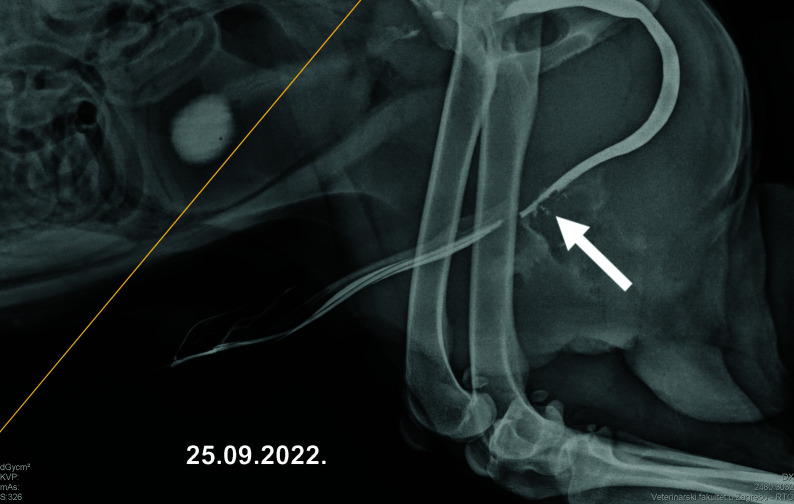
Leakage of contrast during positive contrast-retrograde cystography (shown with an arrow).

We present a case of a two-year-old male dog, breed chow-chow, who had previously had a cystotomy, but the urinary stones had not been completely removed, which lead to a urethral decubital lesion caused by an entrapped urethral stone. Such obstruction ultimately leads to decubital perforation and fistula formation. In this case, the initial surgical attempt had failed, leading to an even worse tissue condition found in our exploration. About ten days after the first surgical procedure performed in a private outpatient clinic, the dog was admitted to our clinic, and a positive-contrast retrograde cystography (Figure 1) confirmed a urethral fistula. At this point, the dog was still not able to urinate on its own, and the bladder was regularly emptied by catheterization or cystocentesis, whereas the scrotum was enlarged with the urine accumulation.

**Figure 2:**
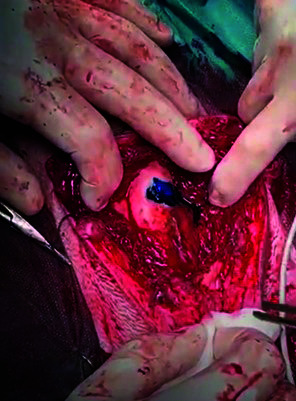
Application of methylene blue through the urinary catheter for the localization of the urethral fistula.

In the first part of the surgical procedure, orchidectomy was performed accompanied by scrotal ablation. Orchidectomy was mandatory considering that cystine urinary stones can cause androgen-dependent cystinuria. Methylene blue was applied via a urinary catheter to detect the exact location of the urethral fistula (Figure 2). The fistula drained into the inflammatory pseudoincapsulated periurethral collection. Fibrous tissue was debrided, and pseudocapsule was excised. The tissue around the fistulous opening was callous and thick, poorly vascularized, and incompliant.

Due to the confirmed effects of MFAT containing MSC and PRP in promoting wound healing, regeneration, and neovascularization, we decided to combine the aspects of regenerative medicine focused on treatment with stem cells obtained from the patient's abdominal fat tissue. The urethral defect was closed with simple interrupted resorptive sutures. At that point, a sample of abdominal adipose tissue from the dog was collected, measuring approximately 8 cm x 8 cm x 2 cm. The fat tissue was finely minced using scissors and an 11-blade at a separate workstation, and introduced into the Lipogems® ortho kit (Lipogems International SpA, Milan, Italy) for the extraction of MFAT, with the system pre-filled with saline solution to avoid air presence.

**Figure 3:**
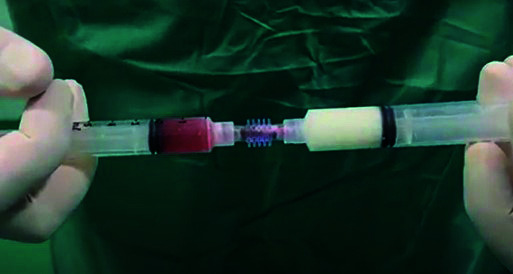
Combining microfragmented adipose tissue with platelet-rich plasma for the application of the combined product into the urethral defect.

The system was then agitated three times for 15 seconds each, followed by rinsing with saline solution. Finally, 5 mL of MFAT was obtained and drawn into a syringe. Subsequently, 15 mL of the dog's venous blood was collected and introduced into the Arthrex ACP® Double-Syringe System (Arthrex, Munich, Germany). After centrifugation for 5 minutes at 1500 rpm, 2 mL of PRP was acquired. This PRP was combined with the previously obtained MFAT using the inter-syringe processing to create the final product, a mixture of MFAT and PRP, intended for therapeutic use (Figure 3). The mixture was then applied through a 16-gauge needle, around the sutures in the area of the urethral defect closure, with care being taken to avoid excessive tissue pressure. Tissue was easily compliant for an amount of 3 mL MFAT/PRP mixture, and the rest was used to freely “shower” the external urethral wall. This meticulous method of combining MFAT with PRP harnesses the regenerative properties of both components, offering a promising approach for canine medical treatments.

**Figure 4:**
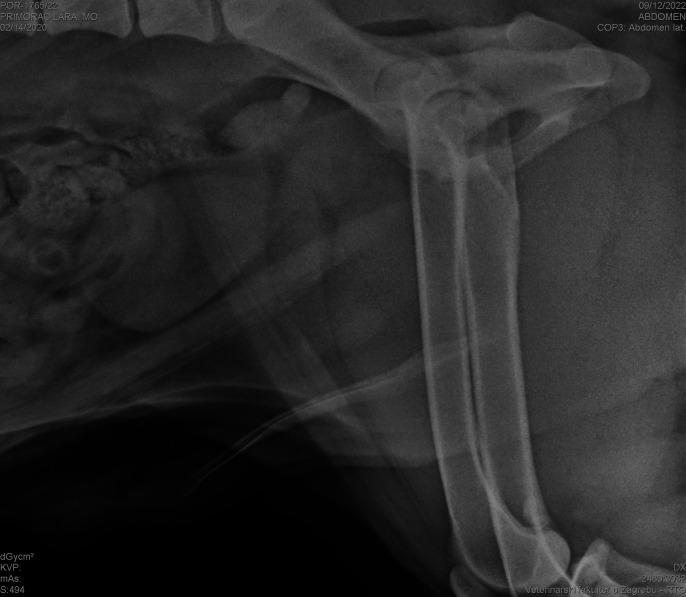
Positive contrast-retrograde cystography showing no contrast leakage. The adipose tissue harvesting site healed uneventfully. One year after surgery, the patient urinated independently and was asymptomatic.

A urinary catheter was left *in situ* for the next 10 days to relieve the urethra and facilitate the healing process. Upon its removal, the patient urinated independently. Three months after the surgery, a positive cystourethrography showed that the urethra was completely healed, with no signs of leakage or strictures (Figure 4).

## Discussion

Urethral defects often present a clinical challenge, especially if the first surgical attempt fails. Multiple factors interact in the process of normal urethral healing, such as intercellular signaling, resident and migrating cell secretome, cytokines, and growth factors (7). Urethral defects have especially been an object of cell-based regeneration strategies, with various forms of regenerative medicine attempts (8-11).

Xue et al showed a clear superiority of cell-enriched scaffold treatment in comparison with non-cell-enriched scaffold repairs (9). This positive effect is achieved mostly by reducing inflammation at the repair site and promoting re-vascularization, which led to less scarring (12).

Stem cells have long been used in orthopedics, particularly in the treatment of osteoarthritis, where they have not only demonstrated excellent clinical outcomes but have also facilitated the restoration of glycosaminoglycan levels in cartilage, which underscores their significant regenerative potential (13-18). Furthermore, MSCs have not only been used for local administration but also for systemic application in conditions such as sarcoidosis or COVID-19 (19,20).

The therapeutic potential of MSCs in urethral repair processes hinges on their ability to modulate the local microenvironment. Stem cells not only differentiate into required cell types but also secrete various paracrine factors that can mitigate inflammation and enhance healing (21). The capacity of these cells to recruit other regenerative cells, support angiogenesis, and suppress fibrotic scarring can play a crucial role in improving surgical outcomes in urethral reconstructions (6). This biological activity underscores the importance of integrating advanced cellular therapies in strategies aimed at repairing complex urethral injuries.

Second-attempt repairs, poor tissue quality and compliance, and abundant scarification are highly predictive of surgical reconstruction failure. The presented case marks the first instance of using MSC as an adjunct therapy in treating a urethral defect in a dog. This innovative approach of applying autologous MFAT and PRP directly to the site of a surgically treated urethral defect offers an effective treatment strategy, without ethical issues and in a fairly simple one-stage procedure. Furthermore, the local delivery of MSC and growth factors from MFAT and PRP can enhance the healing environment by providing high concentrations of growth factors and cytokines directly at the injury site, which can significantly increase the chances of successful tissue regeneration and recovery (22). The same method is often used for treating knee osteoarthritis with excellent clinical results (23). This strategy promotes a more robust healing process, potentially leading to quicker recovery times and better overall health outcomes for the patient. Similar findings were observed by Dimova et al in Crohn's disease rectovaginal fistula repair by using MFAT in combination with a surgical approach (24).

While this case pertains to veterinary medicine, the underlying rationale and biological mechanisms are equally applicable to human medical treatments, which suggests a promising avenue for future clinical applications in urethral repair.

In conclusion, while this innovative application of MFAT and PRP in urethral defect treatment shows promise, the results of this case study must be validated through further research. Controlled clinical trials involving larger canine populations, and eventually human subjects, are necessary to standardize treatment protocols and confirm the efficacy and safety of this approach. Long-term studies focusing on the outcomes and potential complications of this treatment would provide deeper insights into its effectiveness. Additionally, research into the cost-effectiveness and practical application of this therapy could pave the way for its integration into standard medical practice. Future collaborations across veterinary and medical fields are essential to explore the translational potential of this therapy from animals to humans, ensuring the approach is both effective and safe across different species.

## References

[1] Hardie EM Kyles AE Management of ureteral obstruction. Vet Clin North Am Small Anim Pract 2004 34 989 1010 10.1016/j.cvsm.2004.03.008 15223212

[2] Defarges A, Michelle E, Dunn M, Berent A. Urolithiasis in small animals. In: Bruyette DS, ed. Clinical small animal internal meidicine. Wiley-Blackwell; 2020:1123-1130.

[3] Langston C Gisselman K Palma D McCue J Diagnosis of urolithiasis. Compend Contin Educ Vet 2008 30 447 50, 452-4, quiz 455 18833542

[4] Johnston SA, Tobias KM. Veterinary surgery: small animal expert consult. 2nd ed. Saunders; 2017.

[5] McLoughlin MA Complications of lower urinary tract surgery in small animals. Vet Clin North Am Small Anim Pract 2011 41 889 913 10.1016/j.cvsm.2011.07.001 21889691

[6] Jin Y Zhao W Yang M Cell-based therapy for urethral regeneration: a narrative review and future perspectives. Biomedicines 2023 11 2366 10.3390/biomedicines11092366 37760808 PMC10525510

[7] Farzamfar S Elia E Chabaud S Naji M Bolduc S Prospects and challenges of electrospun cell and drug delivery vehicles to correct urethral stricture. Int J Mol Sci 2022 23 10519 10.3390/ijms231810519 36142432 PMC9502833

[8] Chen C Zheng S Zhang X Transplantation of amniotic scaffold-seeded mesenchymal stem cells and/or endothelial progenitor cells from bone marrow to efficiently repair 3-cm circumferential urethral defect in model dogs. Tissue Eng Part A 2018 24 47 56 10.1089/ten.tea.2016.0518 28363256

[9] Xue JD Gao J Fu Q Feng C Xie H Seeding cell approach for tissue-engineered urethral reconstruction in animal study: A systematic review and meta-analysis. Exp Biol Med (Maywood) 2016 241 1416 28 10.1177/1535370216640148 27022134 PMC4994921

[10] Orabi H AbouShwareb T Zhang Y Yoo JJ Atala A Cell-seeded tubularized scaffolds for reconstruction of long urethral defects: a preclinical study. Eur Urol 2013 63 531 8 10.1016/j.eururo.2012.07.041 22877501 PMC3554849

[11] Chan YY Bury MI Yura EM Hofer MD Cheng EY Sharma AK The current state of tissue engineering in the management of hypospadias. Nat Rev Urol 2020 17 162 75 10.1038/s41585-020-0281-4 32024995

[12] Fu Q Cao YL Tissue engineering and stem cell application of urethroplasty: from bench to bedside. Urology 2012 79 246 53 10.1016/j.urology.2011.08.043 22014966

[13] Primorac D Molnar V Rod E Knee osteoarthritis: a review of pathogenesis and state-of-the-art non-operative therapeutic considerations. Genes (Basel) 2020 11 854 10.3390/genes11080854 32722615 PMC7464436

[14] Hudetz D Borić I Rod E Early results of intra-articular micro-fragmented lipoaspirate treatment in patients with late stages knee osteoarthritis: a prospective study. Croat Med J 2019 60 227 36 10.3325/cmj.2019.60.227 31187950 PMC6563172

[15] Hudetz D Borić I Rod E The effect of intra-articular injection of autologous microfragmented fat tissue on proteoglycan synthesis in patients with knee osteoarthritis. Genes (Basel) 2017 8 270 10.3390/genes8100270 29027984 PMC5664120

[16] Hudetz D, Jeleč Ž, Rod E, Borić I, Plečko M, Primorac D. The future of cartilage repair BT - personalized medicine in healthcare systems: legal, medical and economic implications. In: Bodiroga-Vukobrat N, Rukavina D, Pavelić K, Sander GG, eds. Personalized medicine in healthcare systems. Springer International Publishing; 2019:375-411.

[17] Borić I Hudetz D Rod E A 24-Month follow-up study of the effect of intra-articular injection of autologous microfragmented fat tissue on proteoglycan synthesis in patients with knee osteoarthritis. Genes (Basel) 2019 10 1051 10.3390/genes10121051 31861180 PMC6947241

[18] Zenić L Polančec D Hudetz D Medicinal signaling cells niche in stromal vascular fraction from lipoaspirate and microfragmented counterpart. Croat Med J 2022 63 265 72 10.3325/cmj.2022.63.265 35722695 PMC9284019

[19] Pavelic E Matišić V Molnar V Treatment of pulmonary sarcoidosis using allogenic bone marrow-derived mesenchymal stem cell therapy is safe: a case report. Int J Med Sci Clin Res Stud. 2022 02 512 5 10.47191/ijmscrs/v2-i6-12

[20] Primorac D Stojanović SStipić, et alCompassionate mesenchymal stem cell treatment in a severe COVID-19 patient: a case report. Croat Med J 2021 62 288 96 10.3325/cmj.2021.62.288 34212566 PMC8275939

[21] Murphy MB Moncivais K Caplan AI Mesenchymal stem cells: environmentally responsive therapeutics for regenerative medicine. Exp Mol Med 2013 45 e54 54 10.1038/emm.2013.94 24232253 PMC3849579

[22] Molnar V Pavelić E Vrdoljak K Mesenchymal stem cell mechanisms of action and clinical effects in osteoarthritis: a narrative review. Genes (Basel) 2022 13 949 10.3390/genes13060949 35741711 PMC9222975

[23] Molnar V Pavelić E Jeleč Ž Results of treating mild to moderate knee osteoarthritis with autologous conditioned adipose tissue and leukocyte-poor platelet-rich plasma. J Pers Med 2022 13 47 10.3390/jpm13010047 36675708 PMC9864413

[24] Dimova A Erceg Ivkošić I Brlek P Novel approach in rectovaginal fistula treatment: combination of modified martius flap and autologous micro-fragmented adipose tissue. Biomedicines 2023 11 2509 10.3390/biomedicines11092509 37760949 PMC10525900

